# Genome-wide identification of cyclin-dependent kinase (CDK) genes affecting adipocyte differentiation in cattle

**DOI:** 10.1186/s12864-021-07653-8

**Published:** 2021-07-12

**Authors:** Cuili Pan, Zhaoxiong Lei, Shuzhe Wang, Xingping Wang, Dawei Wei, Xiaoyan Cai, Zhuoma Luoreng, Lei Wang, Yun Ma

**Affiliations:** 1 School of Agriculture, Ningxia University, Yinchuan, 750021 China; 2 Key Laboratory of Ruminant Molecular and Cellular Breeding, Ningxia Hui Autonomous Region, Ningxia University, Yinchuan, 750021 China; 3 College of Life Sciences, Xinyang Normal University, Xinyang, 464000 Henan China

**Keywords:** CDK gene family, Bovine, Adipocytes differentiation, Collinearity analysis, Gene expression pattern

## Abstract

**Background:**

Cyclin-dependent kinases (CDKs) are protein kinases regulating important cellular processes such as cell cycle and transcription. Many CDK genes also play a critical role during adipogenic differentiation, but the role of CDK gene family in regulating bovine adipocyte differentiation has not been studied. Therefore, the present study aims to characterize the CDK gene family in bovine and study their expression pattern during adipocyte differentiation.

**Results:**

We performed a genome-wide analysis and identified a number of CDK genes in several bovine species. The CDK genes were classified into 8 subfamilies through phylogenetic analysis. We found that 25 bovine CDK genes were distributed in 16 different chromosomes. Collinearity analysis revealed that the CDK gene family in *Bos taurus* is homologous with *Bos indicus*, *Hybrid-Bos taurus*, *Hybrid Bos indicus*, *Bos grunniens* and *Bubalus bubalis*. Several CDK genes had higher expression levels in preadipocytes than in differentiated adipocytes, as shown by RNA-seq analysis and qPCR, suggesting a role in the growth of emerging lipid droplets.

**Conclusion:**

In this research, 185 CDK genes were identified and grouped into eight distinct clades in Bovidae, showing extensively homology. Global expression analysis of different bovine tissues and specific expression analysis during adipocytes differentiation revealed *CDK4*, *CDK7*, *CDK8*, *CDK9* and *CDK14* may be involved in bovine adipocyte differentiation. The results provide a basis for further study to determine the roles of CDK gene family in regulating adipocyte differentiation, which is beneficial for beef quality improvement.

**Supplementary Information:**

The online version contains supplementary material available at 10.1186/s12864-021-07653-8.

## Background

With the improvement of people’s living standards, high-quality beef is becoming more popular. A crucial factor that affects the flavor, freshness, juiciness, tenderness and color in beef is the intramuscular fat (IMF) content. The latter plays an key role in improving taste and nutritional value, therefore it is important to shed light on the molecular mechanism of IMF deposition in cattle. Adipogenic differentiation is a complex process that includes mitotic clonal expansion (MCE) and terminal adipocyte differentiation [1–3]. Promotion of the cell cycle and maintenance of MCE, necessary for the terminal adipocyte differentiation, depend on cyclin-dependent kinases [4]. In turn, adipocyte terminal differentiation requires exiting from the mitotic cycle and entry into a permanent state of G1 arrest, together with expression of adipogenic phenotypic genes [1]. PPARγ C/EBPα and C/EBPβ are the key transcription factors in the regulatory network that functions during both MCE and terminal adipocyte differentiation [5]. During MCE, C/EBPβ can transactivate the expression of multiple cell cycle-related genes and also facilitate adipocyte hyperplasia by delaying the expression of *C/EBPα* and *PPARγ* [5]. When adipocytes accumulate above a threshold, terminal differentiation is initiated, and several genes are expressed to directly or indirectly regulate *C/EBPα* and *PPARγ*. Then activated PPARγ and C/EBPα regulate the expression of adipogenic phenotypic genes, e.g., *FABP1*, *FABP4*, *LPL*, *aP2*, *CAP* and *Perilipin*, leading to lipid droplet formation [5–7]. Thus, during adipogenic differentiation there is a variety of genes in families such as *KLFs*, *SMADs*, *RARs*, *DGATs SREBPs, CDKs* and others, that function synergistically [8, 9]. Herein, we focus on the regulatory mechanism of the CDK gene family in adipocyte differentiation.

CDKs are a large family of serine/threonine protein kinases. They were first discovered in the context of regulation of the cell cycle and have diverse functions in eukaryotes, like mRNA processing, regulation of transcription [10–13]. Recently, they have been shown to regulate adipocyte differentiation and lipid droplet formation by phosphorylating a series of associated transcription factors or adipocyte-specific genes. When the member of the CDK family genes *CDK6* was targeted by miR107 in adipocytes, *Notch* and its downstream gene *Hes1* were downregulated and this caused inhibition of glucose uptake and triglyceride synthesis in adipocytes [14]. MAPK and CDK2/cyclinA sequentially activate C/EBPβ by maintaining the phosphorylated state of Thr188 during MCE progression and adipocytes terminal differentiation [15]. Insulin activates the CCND3-CDK4 complex which in turn phosphorylates the insulin receptor IRS2 at Ser388; this maintains an active insulin signaling pathway in adipocytes, eventually promoting de novo lipid synthesis [16]. In addition, CDK4 can phosphorylate Rb to release E2F, leading to preadipocyte proliferation as well as phosphorylation of PPARγ which regulates the terminal differentiation of adipocytes [17]. CDK5 can reduce the insulin sensitivity of adipocytes by phosphorylating PPARγ at Ser273, and inhibition of this phosphorylation promotes browning and thermogenesis in white adipose tissue [18]. The CDK7 complex can inactivate PPARγ through PPARγ-S112 phosphorylation and inhibit adipogenesis [19]. CDK8 inhibits adipogenesis by phosphorylating the serine residues of SREBP-1c, leading to its ubiquitination and degradation [20]. These findings inspired our curiosity and guided our exploration of the impact of the CDK gene family on bovine adipocyte differentiation.

The expression patterns and regulatory mechanisms of CDK genes in bovine adipocytes have not been systematically studied and elucidated. Therefore, in the present study we aimed to first detect the CDK gene family in the bovine genome and to elucidate their physicochemical properties and structural features together with detailed classification, phylogenetic and functional analyses. In addition, to identify essential members of CDK family that affect adipogenic differentiation, we performed expression pattern analysis by RNA sequencing and qPCR. Our study provides deep insights into CDK genes that influence adipogenic differentiation and is beneficial for future studies to improve IMF in the context of bovine breeding.

## Results

### Identification of CDK family members

To identify the CDK family members, 59 verified CDK amino acid sequences were used as the query. These belonged to cattle (*Bos taurus,* 8), human (*Homo sapiens,* 26) and mouse (*Mus musculus,* 25). Query sequences were used for genome-wide detection of homologous sequences in *Bos taurus*, *Bos indicus*, *Bos grunniens*, *Hybrid-Bos indicus*, *Hybrid-bos taurus*, *Bos mutus*, *Bison bison bison*, and *Bubalus bubalis*. In *Bos taurus*, 25 non-redundant CDK protein sequences were identified that included CDK1–10, CDK11B, CDK12–20 and CDKL1–5 (Table 1). CDK family proteins were also recognized in *Bos grunniens* (22), *Hybrid-Bos indicus* (21), *Hybrid-bos taurus* (22), *Bos mutus* (24), *Bison bison bison* (22), *Bos indicus* (25) and *Bubalus bubalis* (24) (Additional file 1). Protein sequences of all CDKs are provided in Additional file 2. A newly identified member of the CDK family was found in *Hybrid-bos taurus*, Transcript ENSBIXT00000049337, and was named as *CDK20* following sequence similarity and collinearity.

**Table 1:** Characteristics of genome-wide identified CDK family members in *Bos taurus*

Protein Name	Gene ID	Transcript ID	pI	Mw/Da	Amino acids	Description
CDK1	ENSBTAG00000010109	ENSBTAT00000013337	8.38	34,025.40	297	cyclin-dependent kinase 1
CDK2	ENSBTAG00000004021	ENSBTAT00000005252	8.79	33,873.46	298	cyclin-dependent kinase 2
CDK3	ENSBTAG00000010509	ENSBTAT00000013885	8.13	34,805.48	305	cyclin-dependent kinase 3
CDK4	ENSBTAG00000007160	ENSBTAT00000009420	6.51	33,646.73	303	cyclin-dependent kinase 4
CDK5	ENSBTAG00000007766	ENSBTAT00000010212	7.57	33,288.47	292	cyclin-dependent kinase 5
CDK6	ENSBTAG00000044023	ENSBTAT00000061349	6.22	37,014.40	326	cyclin-dependent kinase 6
CDK7	ENSBTAG00000011046	ENSBTAT00000014667	8.67	38,946.26	346	cyclin-dependent kinase 7
CDK8	ENSBTAG00000016737	ENSBTAT00000022252	8.72	53,282.71	464	cyclin-dependent kinase 8
CDK9	ENSBTAG00000004695	ENSBTAT00000006162	9.04	42,747.58	372	cyclin-dependent kinase 9
CDK10	ENSBTAG00000033333	ENSBTAT00000047400	9.16	41,046.93	361	cyclin-dependent kinase 10
CDK11B	ENSBTAG00000010737	ENSBTAT00000014227	5.34	89,901.85	771	cyclin-dependent kinase 11B
CDK12	ENSBTAG00000013238	ENSBTAT00000002005	9.54	140,641.60	1264	cyclin-dependent kinase 12
CDK13	ENSBTAG00000001528	ENSBTAT00000002003	9.71	164,717.14	1512	cyclin-dependent kinase 13
CDK14	ENSBTAG00000048664	ENSBTAT00000068321	9.06	53,169.98	470	cyclin-dependent kinase 14
CDK15	ENSBTAG00000055073	ENSBTAT00000086547	6.68	45,011.42	405	cyclin-dependent kinase 15
CDK16	ENSBTAG00000016769	ENSBTAT00000022303	7.23	55,758.68	496	cyclin-dependent kinase 16
CDK17	ENSBTAG00000001510	ENSBTAT00000077282	9.1	59,563.16	523	cyclin-dependent kinase 17
CDK18	ENSBTAG00000012673	ENSBTAT00000085187	9.26	54,126.19	471	cyclin-dependent kinase 18
CDK19	ENSBTAG00000007288	ENSBTAT00000009583	8.66	56,685.13	500	cyclin-dependent kinase 19
CDK20	ENSBTAG00000015171	ENSBTAT00000020188	6.06	38,546.53	346	cyclin-dependent kinase 20
CDKL1	ENSBTAG00000004780	ENSBTAT00000036046	9.08	40,735.16	352	cyclin-dependent kinase like 1
CDKL2	ENSBTAG00000014038	ENSBTAT00000031574	8.76	64,289.09	569	cyclin-dependent kinase like 2
CDKL3	ENSBTAG00000010979	ENSBTAT00000014574	9.37	67,477.82	591	cyclin-dependent kinase like 3
CDKL4	ENSBTAG00000024044	ENSBTAT00000033135	8.88	39,465.72	342	cyclin-dependent kinase like 4
CDKL5	ENSBTAG00000007428	ENSBTAT00000076996	9.56	107,236.16	960	cyclin-dependent kinase like 5

*Mw* Molecular weight, *pI* Isoelectric point. The gene ID and transcript ID can be referred in the Ensembl database (http://asia.ensembl.org/index.html)

The amino acid lengths of the 25 cattle CDK proteins ranged from 292 (CDK5) to 1512 (CDK13), whereas molecular weight (Mw) ranged from 33,288.47 to 164,717.14 Da, consistent with protein length. The isoelectric point (pI) of most CDK family proteins was higher than 8.0, as they contained more basic amino acids than acidic amino acids. However, CDK5 and CDK16 were neutral, with pIs 7.57 and 7.23, respectively. Also, five proteins were acidic (CDK4, CDK6, CDK11B, CDK15 and CDK20) with pIs ranging from 5.34 to 6.68. All 25 CDK proteins contained the Serine/Threonine Kinase (STK) conserved domain (Additional file 3).

### Structural features of bovine CDK family members

**Fig. 1:**
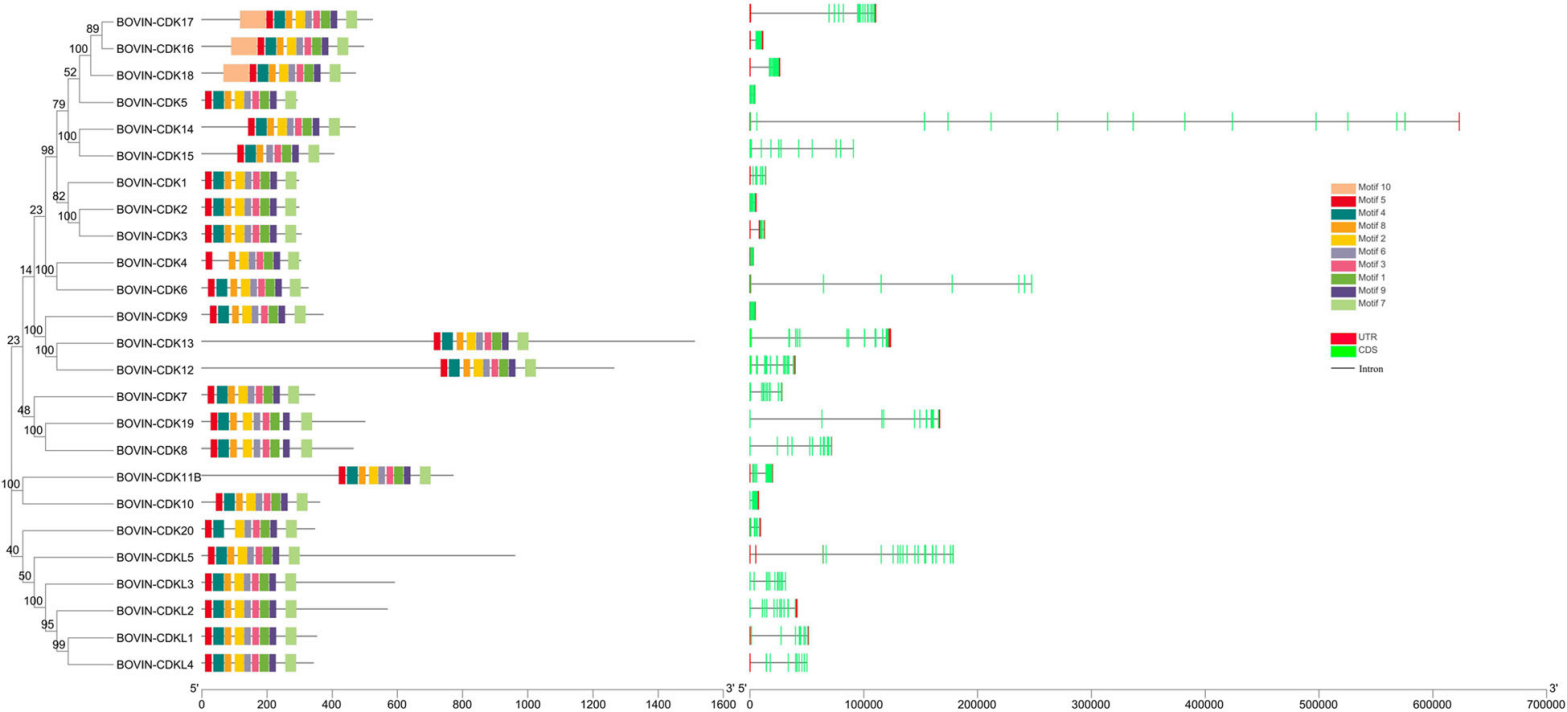
Characterization of the identified CDK proteins and genes in Bos taurus. The phylogenetic tree (left) was constructed by the Neighbor Joining method. Amino acid sequences (middle), where conserved motifs are indicated as rectangles with different colors. Gene structure map (right), where green rectangle, black line and red rectangle represent CDS, intron and UTR, respectively

To explore the structural characteristics of bovine CDK proteins and genes, the conserved motifs and gene structures were projected based on their phylogenetic relationships (Fig. 1). CDKs of cattle clustered into three main subfamilies according to the evolutionary clades. One subfamily contains 6 members CDKL1–5 and CDK20, another contains CDK10 and CDK11B, whereas the rest belongs to the third subfamily. All the CDK family proteins shared six conserved domains termed motifs 1, 3, 5, 6, 7, and 9, formed by 29, 21, 21, 21, 21 and 21 amino acids, respectively (Additional file 4). A small branch in the third subfamily formed by CDK16, CDK17 and CDK18, has all ten motifs. CDK4, CDK15 and CDK20 all have eight motifs, since CDK4 lacks 4 and 10, CDK15 lacks 2 and 10, and CDK20 lacks 4 and 10. The remaining CDK proteins comprise nine motifs lacking CDK10, which indicates they all have the same conserved patterns.

Introns, coding sequences (CDS) and untranslated regions (UTR) were variable among the CDK gene family. For instance, the length of CDK genes ranged from 3599 nt (*CDK4*) to 678,562 nt (*CDK14*), mainly due to intron variation. The number of CDS varied from 7 to 17, and the length and layout of 3’UTR and 5’UTR were also variable in the noncoding areas. Despite this variability in CDS, introns and UTRs, we discovered that CDK family members in the same evolutionary branch tend to show similar gene structures and conserved patterns in motifs.

### Phylogenetic relationship of CDK proteins in different organisms

**Fig. 2:**
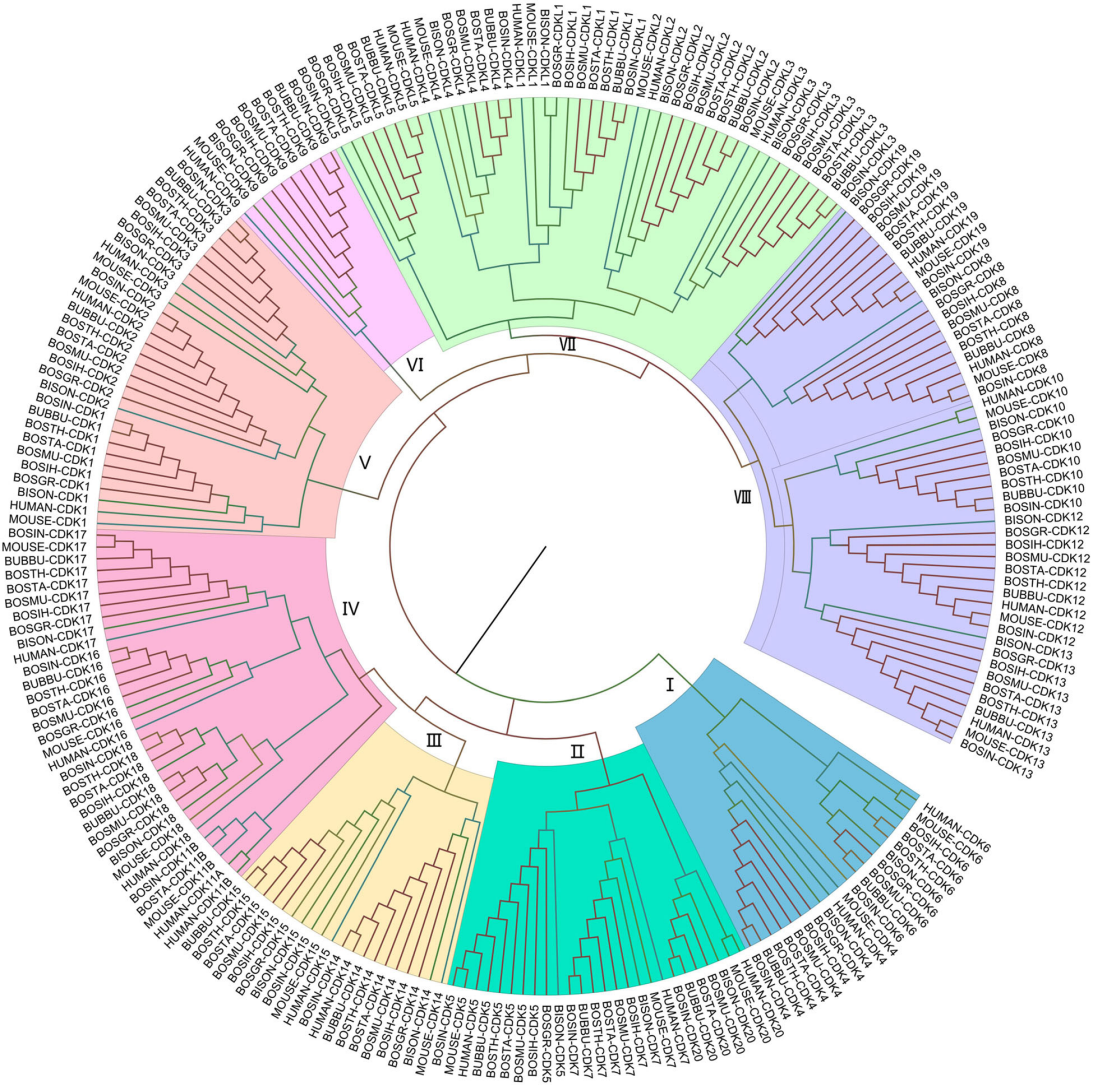
Phylogenetic Neighbor-Joining (NJ) tree of CDK proteins from ten organisms. Identified CDKs in Bos taurus (BOSTA), *Bos grunniens* (BOSGR), *Hybrid-Bos Indicus* (BOSIH), *Hybrid-Bos taurus* (BOSTH), *Bos mutus* (BOSMU), *Bison bison bison* (BISOM), *Bos indicus* (BOSIN) and *Bubalus bubalis* (BUBBU) together with verified CDKs from *Homo sapiens* (HUMAN) and *Mus musculus* (MOUSE). CDK proteins are grouped into eight clusters (I-VII) shown as different colors

To assess the CDK proteins evolutionary relationships between cattle and other organisms, we conducted a phylogenetic analysis that included eight Bovinae species (*Bos taurus, Bos indicus, Bos grunniens*, *Hybrid-Bos indicus*, *Hybrid-bos taurus*, *Bos mutus*, *Bison bison bison*, and *Bubalus bubalis*) and *Homo sapiens* and *Mus musculus.* CDK proteins in human and mouse were also included since they have been studied extensively as model organisms. From these ten organisms, 236 amino acid sequences were aligned to generate a nonrooted Neighbor-Joining (NJ) tree (Fig. 2). Phylogenetic analyses revealed a clustering of CDK family proteins into eight major clades.

### Chromosomal distribution and collinearity analysis of CDK genes

**Fig. 3:**
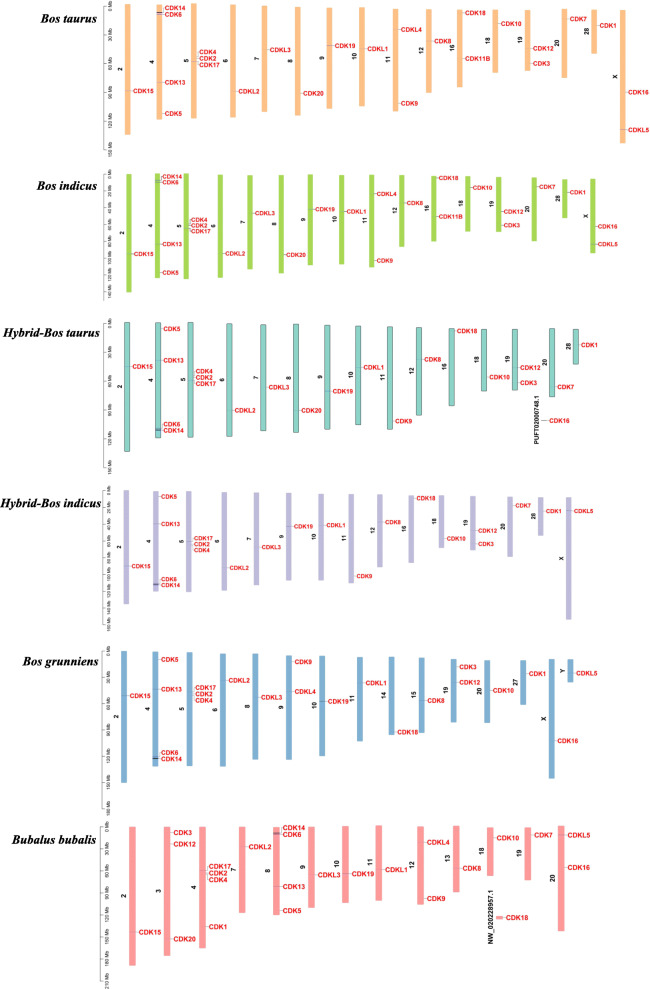
Chromosomal distribution of CDK genes. The black font on the left represents chromosome numbers and the red font on the right represents CDK genes

CDK genes were mapped on the chromosomes of six Bovinae species; 25 bovine CDK genes distribute on 16 chromosomes (Fig. 3). The CDK genes of cattle have a similar distribution as those in *Bos indicus*. However, the order of *CDK14* (7.94–8.62 Mb), *CDK6* (9.94–10.19 Mb), *CDK13* (80.95–81.08 Mb) and *CDK5* (113.630–113.634 Mb) in *Bos taurus* Chr 4 was opposite from that in *Hybrid-Bos indicus*, *Hybrid-bos taurus* and *Bos grunniens*. In *Bos taurus, CDK4*, *CDK2* and *CDK17* were three tandem genes located at 29.66–29.69 Mb, 29.66–29.69 Mb and 29.72–29.92 Mb on Chr 5, whereas in *Bos grunniens*, *Hybrid-Bos indicus* Chr 5 and *Bubalus bubalis* Chr 4, the arrangement of these three genes was reversed. In addition, compared with *Bos taurus*, several genes were missing in *Bos grunniens* (*CDK7*, *CDK11B* and *CDK20*), *Hybrid-Bos indicus* (*CDK11B*, *CDK16*, *CDK20* and *CDKL4*), *Hybrid-bos taurus* (*CDK11B*, *CDK20*, *CDKL4* and *CDKL5*), and *Bubalus bubalis* (*CDK11B*). Also, *CDKL5* is located at chromosome X in *Bos taurus*, whereas it is in chromosome Y in *Bos grunniens.*

**Fig. 4:**
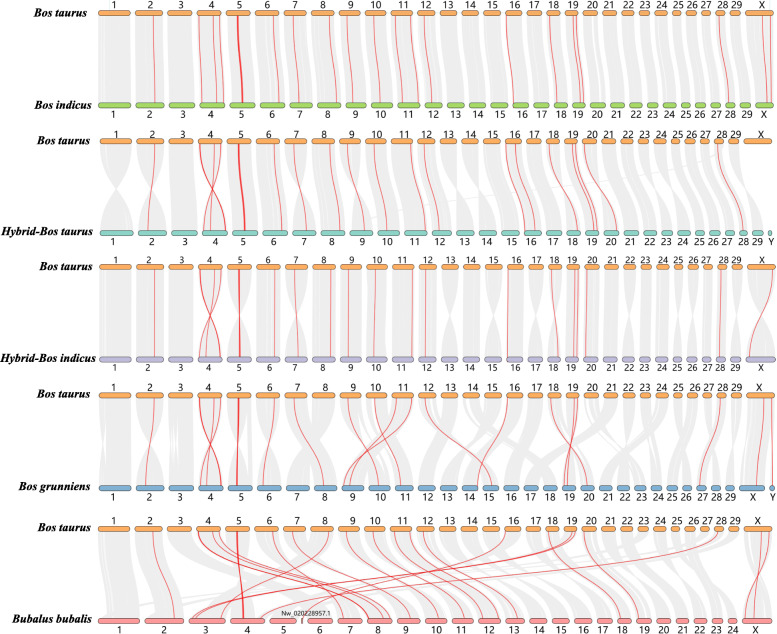
Collinearity analysis of CDK genes between cattle and other organisms. Syntenic genes pairs are linked by grey lines whereas syntenic CDK genes are shown as red lines

Genome collinearity analysis resulted in the identification of pairs of collinear genes between *Bos taurus* and *Bos indicus* (31,691), *Hybrid-Bos indicus* (34,495), *Hybrid-bos taurus* (33,570), *Bos grunniens* (32,378) and *Bubalus bubalis* (33,327), respectively (Fig. 4). There is a one-to-one correspondence between chromosomes of *Bos taurus* and *Hybrid-Bos indicus*, *Hybrid-bos taurus*, *Bos indicus* and *Bos grunniens*. A large chromosome homology also existed between cattle (2 *N* = 60) and buffalo (2 *N* = 50), although the chromosome number is different in the two species. The syntenic blocks revealed syntenic relationships in the CDK gene family between cattle and the other five species in Bovinae (Table 2).

**Table 2:** Syntenic relationships of CDK family genes between cattle and the other five species

Gene	*Bos indicus*	*Hybrid-bos taurus*	*Hybrid-Bos Indicus*	*Bos grunniens*	*Bubalus bubalis*
*CDK1*	Y	Y	Y	Y	Y
*CDK2*	Y	Y	Y	Y	Y
*CDK3*	Y	Y	Y	Y	Y
*CDK4*	Y	Y	Y	Y	Y
*CDK5*	Y	Y	Y	Y	Y
*CDK6*	Y	Y	Y	Y	Y
*CDK7*	N	Y	Y	–	Y
*CDK8*	Y	Y	Y	Y	Y
*CDK9*	Y	Y	Y	Y	Y
*CDK10*	Y	Y	Y	Y	Y
*CDK11B*	N	–	–	–	–
*CDK12*	Y	Y	Y	Y	Y
*CDK13*	Y	Y	Y	Y	Y
*CDK14*	Y	Y	Y	Y	Y
*CDK15*	Y	Y	Y	Y	Y
*CDK16*	Y	Y	–	Y	Y
*CDK17*	Y	Y	Y	Y	Y
*CDK18*	Y	Y	Y	Y	Y
*CDK19*	Y	Y	Y	Y	Y
*CDK20*	Y	Y	–	–	Y
*CDKL1*	Y	Y	Y	Y	Y
*CDKL2*	Y	Y	Y	Y	Y
*CDKL3*	Y	Y	Y	Y	Y
*CDKL4*	Y	–	–	Y	Y
*CDKL5*	Y	–	Y	Y	Y

‘Y’ represents the synteny of genes between two species, while ‘N’ means not and ‘-’ means lacking of the gene

### Expression analysis of CDK genes in different tissue

**Fig. 5:**
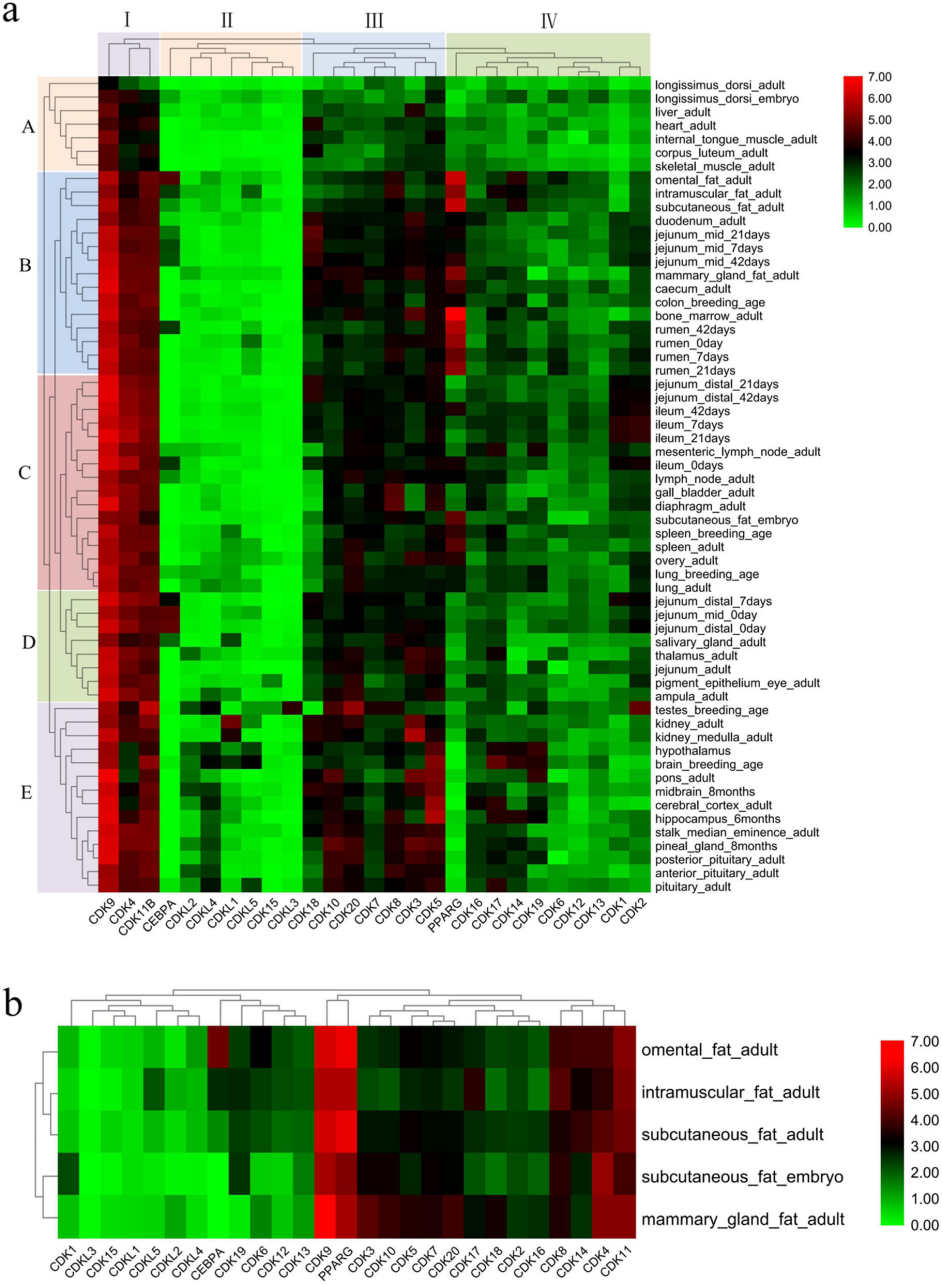
Expression analysis of the CDK gene family in different bovine tissue types. a. Expression analysis of theCDK gene family in 60 bovine tissues. The tissues were classified into 5 groups (A to E) and the 27 genes were classified into 4 groups (I-IV) according to their expression pattern. b. Expression analysis of CDK gene family in 5 bovine fat tissues. The horizontal axis represents 25 *CDKs* and 2 marker genes (*PPARG* and *CEBPA*) of adipocyte differentiation. The vertical axis represents different bovine tissues

The expression patterns of genes could provide important references for their function. To explore the expression patterns of the CDK gene family during adipogenic differentiation, we investigated the relative expression levels in 163 samples of 60 tissue types. The results showed that CDK genes displayed differential expression patterns in diverse tissues (Fig. 5 a), which could be classified into five groups (A to E). *PPARγ* is a marker gene for adipocyte differentiation, and consistent with this, its expression was high in Group B which included omental, intramuscula, subcutaneous and mammary gland fats. According to their expression patterns, the 25 CDK genes could be grouped into four categories. They were all expressed in the 60 tissues, suggesting a broad regulatory role in life activities. Group I (*CDK4*, *CDK9* and *CDK11B*) showed the highest expression levels, followed by Group III (*CDK3*, *CDK5*, *CDK7*, *CDK8*, *CDK10*, *CDK18*, and *CDK20*) and Group IV (*CDK1*, *CDK2*, *CDK6*, *CDK12*, *CDK13*, *CDK14*, *CDK16*, and *CDK17*). The rest of the members were in Group II and their expression was the lowest. Further analysis of the five different fat tissues revealed that *CDK9* was highly expressed in all of them with an expression pattern similar to *PPARγ* (Fig. 5 b).

### Expression analysis of CDK genes in preadipocytes and differentiated adipocytes by RNA-seq

**Fig. 6:**
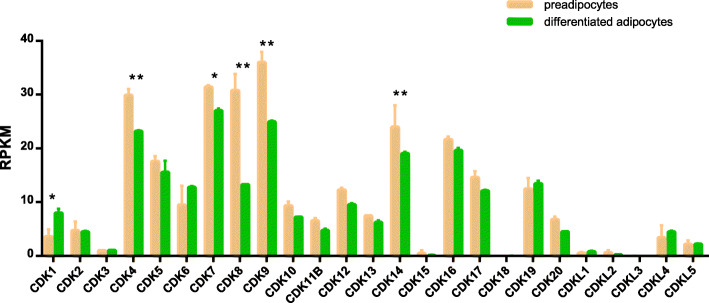
Expression analysis of CDK genes in preadipocytes and differentiated adipocytes by RNA-seq. Error bars were obtained from two measurements. Symbols * and ** above the bars indicate significant differences at 0.05 and 0.01 level between preadipocytes and differentiated adipocytes

As revealed by transcriptome analysis, 25 CDK genes showed an up-regulation trend in preadipocytes compared with differentiated adipocytes, except for *CDK1*, *CDK3*, *CDK6*, *CDK19*, *CDKL1* and *CDKL4* (Fig. 6). *CDK7* displayed high expression, whereas *CDK1* showed low expression in preadipocytes (within the 95% confidence interval). *CDK4*, *CDK8*, *CDK9* and *CDK14* displayed a high expression in preadipocytes (within the 99% confidence interval).

### Expression analysis of CDK genes during adipocyte differentiation by qPCR

**Fig. 7:**
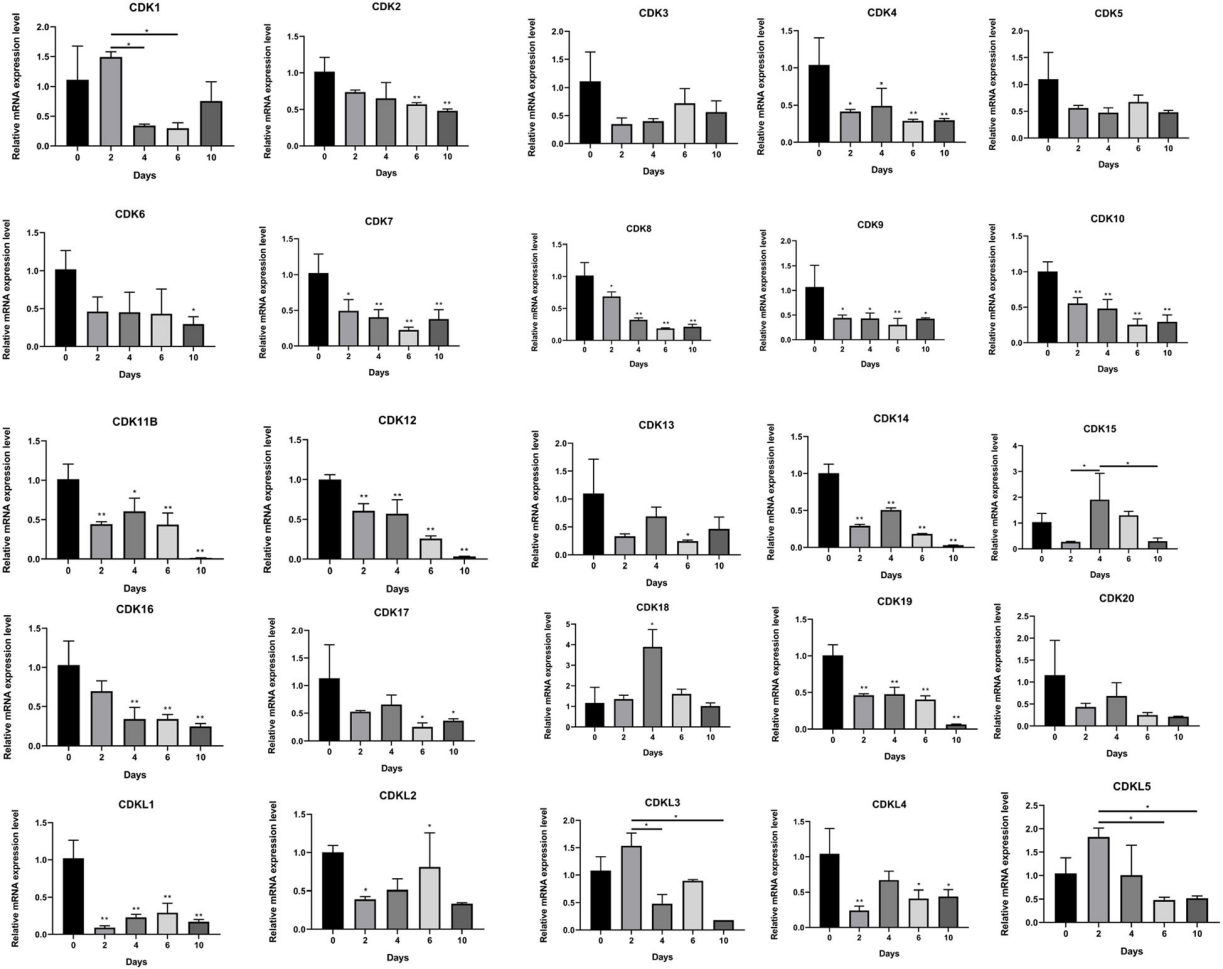
qPCR expression analysis of CDK genes during adipocyte differentiation. Error bars were obtained from three measurements. Symbols * and ** above the bars indicate significant differences at 0.05 and 0.01 level compared with preadipocytes (0 day). These symbols above the lines represent the significance of differences between two labeled pillars

To further explore the expression patterns of the CDK gene family, preadipocytes collected from perirenal adipose tissue of premature calves were induced to differentiate. The results of Oil Red O staining showed that lipid droplet accumulation increased in adipocytes induced for 10 days compared to preadipocytes (Additional file 5), indicating that induction and differentiation was successful. A qPCR analysis was conducted to detect the expression of CDK genes at 0, 2, 4, 6, and 10 days during adipocytes differentiation (Fig. 7). CDK genes showed a relatively high expression in preadipocytes and then decreased as differentiation progressed in addition to *CDK1*, *CDK15*, *CDK18*, *CDKL3* and *CDKL5*. *CDK1*, *CDKL3* and *CDKL5* showed the highest expression on the second day of differentiation whereas the lowest expression points were on the 6th, 8th and 6th day, respectively. *CDK15* and *CDK18* increased with adipocyte differentiation and reached a peak on the fourth day, then decreased.

## Discussion

Cattle is known as an important species for supplying meat. The IMF content directly affects the taste and flavor of beef and it is of great scientific significance to reveal the molecular regulation mechanism of IMF deposition. The CDK gene family encoding functional proteins have been well studied in the regulation of transcription, metabolism and cell differentiation [10–12]. However, research on the role of CDK genes in adipocyte differentiation is limited, especially in Bovidae. Since cattle and several species of Bovidae were sequenced, these resources may serve as reference to explore the evolution and function of CDK genes and in advancing genome science in Bovidae.

### Structural features of bovine CDK family proteins and genes

The activity of proteins depends on their functional motifs and domains [21]. Six conserved amino acid sequences (Motifs 1, 3, 5, 6, 7 and 9) were conserved among all CDK family members in cattle. Highly conserved motifs are usually located in active sites of enzymes and are essential in maintaining their structure, binding to substrate or in catalysis [22, 23]. CDK16–18, a small branch in a subfamily, have all of the ten motifs, pointing to a common function in these three members. It is likely that Motif 10 is not located at the core of the catalytic domain since the other 22 members where it is absent still show kinase activities. In addition to Motif 10, other motifs are absent in CDK4 (Motif 4), CDK15 (Motif 2) and CDK20 (Motif 8) indicating some sequence losses during evolution. CDK20 is a newly identified member in *Hybrid-bos taurus*, and has a conserved STKc domain and nine motifs (Additional file 6). Domain and motif analysis revealed its similarity with the other CDK proteins.

The distribution of CDSs, introns, and UTRs was variable in CDK genes, mainly due to the length and layout of introns and UTRs, whereas gene coding sequences and amino acid sequences were similar. These results suggest that amino acid sequence similarity, especially in conserved motifs, may play essential roles in keeping the kinase function in CDK proteins.

### Phylogenetic relationship of CDK family proteins

Phylogenetic analysis of proteins can provide an in-deep insight for their evolutionary relationships [24]. CDK family proteins were classified into eight major clades. The same member from each species first clustered in one branch, indicating that they were conserved in sequences among the ten species. Initially, Clade I separated, whereas Clade II, III and IV clustered into a subfamily and the remaining clustered into another subfamily. This shows that they have evolved asymmetrically and that these subfamilies are distant. Notably, Clade VII includes all the cyclin-dependent kinases like proteins (CDKL1, CDKL2, CDKL3, CDKL4 and CDKL5), consistent with human study that divided the CDK gene family into CDK and CDKL [12]. As expected, members that are closer evolutionarily clustered together first. For example, *Bos indicus* CDK1 first clustered with that of *Bubalus bubalis,* and then get together with *Hybrid-bos taurus*, *Bos taurus*, *Bos mutus*, *Hybrid-Bos indicus*, *Bos grunniens*, *Bison*, human and mouse*.*

### Collinearity analysis of CDKs in Bovidae

Members of a gene family may distribute in the same or in different chromosomes. These two cases are due to tandem or segmental duplication events, respectively [25–27]. The members of the CDK gene family showed a distribution in 13 to 16 chromosomes in six species of Bovidae, indicating that both segmental and tandem duplication events have occurred. In addition to a pair sex chromosomes (XX/XY), the genomes of *Bos taurus*, *Bos grunniens*, *Hybrid-Bos indicus*, *Hybrid-bos taurus*, and *Bos indicus* consist of 29 autosomes [28, 29], whereas *Bubalus bubalis* has 24 autosomes. The collinearity results showed a one-to-one correspondence between chromosomes of *Bos taurus* and *Hybrid-Bos indicus*, *Hybrid-bos taurus*, *Bos indicus* and *Bos grunniens*, and large homologous chromosomal regions between *Bos taurus* and *Bubalus bubalis*. The arrangement of genes in some syntenic chromosomes may be totally reversed between *Bos taurus* and other species. This may be due to the opposite starting point for chromosome annotation. Since all the chromosomes were homologous in Bovidae, the positions where CDK genes located were either collinear (conserved in the same order) or syntenic (not necessarily in the same order) between each two species except for only a few gene pairs [30]. For example, *CDK7* in Chr 20 and *CDK11B* in Chr 16 did not show synteny between *Bos taurus* and *Bos indicus.* The positions of *CDKL4* and *CDK9* are opposite between *Bos taurus* and *Bos grunniens*, which located at 21.50–21.55 Mb and 98.46–98.47 Mb in Chr 11 of *Bos taurus* and 41.02–41.08 Mb and 7.13–7.14 Mb in Chr 9 of *Bos grunniens.* The deficiency and discrepancies of CDK genes might be caused by the sequence variation and chromosome rearrangement in the process of evolution [31]. In addition, Chr16 of *Bos taurus* showed a syntenic relationship with Chr5 and scaffold NW_020228957.1 of *Bubalus bubalis*, suggesting that NW_020228957.1, which has not been assembled yet, may be a part of *Bubalus bubalis* Chr5. Meanwhile, it was syntenic between CDK18 of *Bos taurus* Chr16 and that in scaffold NW_020228957.1 of *Bubalus bubalis.* In a word, the extensive homology provided rich perspectives for studying the function and evolution of CDK gene family in Bovidae.

### CDK genes affecting adipocyte differentiation

CDK family proteins can regulate adipocyte differentiation by phosphorylating a series of transcription-related factors or adipocyte-specific genes [14–18, 20]. To dissect the expression pattern of this family, the expression of its 25 members in 60 tissue types was analyzed. *CDK4*, *CDK9* and *CDK11B* were highly expressed in four types of fat tissues (omental, intramuscular, subcutaneous and mammary gland) relative to other tissues. Thus, we hypothesized that these three genes may be more relevant to adipose tissue regulation than other members. Indeed, CDK4 can phosphorylate IRS2 and Rb to promote adipogenesis [16, 17], whereas CDK9, a component of positive transcription elongation factor b (P-TEFb), can phosphorylate the C-terminal domain of RNA polymerase II and regulate the transcription of target genes by facilitating transcriptional elongation [32]. In 3 T3-L1 cells, CDK9 increased the adipogenic potential by phosphorylating PPARγ directly and inducing its transcriptional activity [33]. In contrast, although *CDK11B* had similar expression patterns to *CDK4* and *CDK9*, its role in adipogenic differentiation has never been described.

Adipogenic differentiation is a complicated and well-organized process regulated by multiple genes. Analysis of CDK gene family expression patterns during adipocyte differentiation is essential to explore its role. *CDK4*, *CDK7*, *CDK8* and *CDK9* were highly expressed in preadipocytes, suggesting a role in targeting newly generated lipid droplets. *CDK1*, *CDKL3* and *CDKL5* reached the highest expression on the second day, whereas *CDK15* and *CDK18* peaked on the fourth day, indicating that they may also play regulatory roles during adipocyte differentiation. The role of *CDK1*, *CDK4*, CDK7, *CDK8* and *CDK9* in adipocyte differentiation has been studied [16, 17, 19, 20, 33–37] but they need to be explored in more detail, whereas *CDK15*, *CDK18*, *CDKL3* and *CDKL5* have not been studied.

Interestingly, the expression trends of some of the members were inconsistent between RNA-seq and qPCR validation tests. For instance, RNA-seq analysis showed no significant differences in the expression of *CDK2*, *CDK6*, *CDK10*, *CDK11B*, *CDK12*, *CDK16*, *CDK17*, *CDK19* and *CDKL1*, but qPCR showed significant down-regulation. This may have been caused by an insufficient number of samples and/or the different sample source used for qPCR. RNA-seq samples were separated from inguinal subcutaneous fat of two 1 year old male Qinchuan cattles, whereas qPCR samples were from perirenal fat of a premature female Holstein calf. Thus, the determination of the function of CDK genes during adipocyte differentiation is complicated and requires an in-depth analysis.

The CDK gene family and the interacting genes formed an integrated network as shown by literature mining using the Agilent Literature Search plug-in of Cytoscape (Additional file 7) [38]. For example, *CDK7* could directly activate *CDK9* to maintain a high expression of *MDM4* and *MDM2* [36, 37]. *MDM2* facilitates adipocyte differentiation through CRTC-mediated activation of *STAT3* [39]. Overall, these results revealed that the CDK gene family interact with each other and other genes, playing non-redundant roles and collectively regulating cell cycle, adipocyte differentiation or lipid metabolism.

## Conclusions

We have conducted a comprehensive genome-wide analysis of the CDK gene family in Bovidae. A total of 185 CDK genes were identified and grouped into eight distinct clades. Collinearity analysis revealed that the CDK gene family is homologous between cattle and other species in Bovinae. Expression analysis and functional prediction indicated that CDK genes may play a significant and complicated role in regulating bovine adipocyte differentiation. These results provide an essential reference for further studies of the CDK gene family in the regulation of bovine adipocyte differentiation.

## Methods

### Ethics statement

Animal experiments were conducted according to the guidelines of the Regulations for the Administration of Affairs Concerning Experimental Animals (Ministry of Science and Technology, China, 2004). All animal protocols were approved by the Animal Ethics Committee of Ningxia University (permit number NXUC20200618) and Zerui ecological breeding farm (permit number ZR20200615). A premature female calf of a Holstein pregnant cow used in the experiment was released and the primary adipocytes were isolated immediately, making all efforts to minimize suffering of the calf. The pregnant cow was not sampled and is still being raised in Zerui ecological breeding farm (Yinchuan, China) after a period of recuperation.

### Genome-wide identification of CDK genes

The genome and annotation of *Bos taurus* (ARS-UCD1.2 assembly), *Bos grunniens* (LU_Bosgru_v3.0 assembly), *Hybrid-Bos indicus* (*Bos indicus*×*Bos taurus*, UOA_Brahman_1 assembly), *Hybrid-bos taurus* (*Bos indicus*×*Bos taurus*, UOA_Angus_1 assembly), *Bos mutus* (BosGru_v2.0 assembly), *Bison bison bison* (Bison_UMD1.0 assembly), *Homo sapiens* (GRCh38.p13 assembly) and *Mus musculus* (GRCm39 assembly) are from Ensembl database (http://useast.ensembl.org/info/data/ftp/index.html); *Bos indicus* (Bos_indicus_1.0 assembly) and *Bubalus bubalis* (UOA_WB_1 assembly) are from NCBI Genome repository (https://www.ncbi.nlm.nih.gov/genome/). To identify all the possible CDK genes in Bovinae, both Hidden Markov Model (HMM) search and Basic Local Alignment Search Tool (BLAST) were performed [40]. A total of 59 reviewed CDKs sequences of bovine (*Bos taurus*)*,* human (*Homo sapiens*) and mouse (*Mus musculus*) were obtained from the UniProt database (https://www.uniprot.org/). These protein sequences were used as seeds to query potential CDK gene family candidates via BLASTP with a threshold of e-value =10^**−** 5^. The HMM of CDKs (PF00069) was downloaded from Pfam (https://pfam.xfam.org/) [41] and HMMER 3.3.1 (http://hmmer.org/) [42] was used to construct HMM profiles in Bovidae for detection of CDK genes with the default setting. Candidate sequences obtained from the two methods were manually checked to confirm the CDK homology. Non-redundant CDK homologs were submitted to NCBI CD-search [43] to verify the presence of the conserved protein domains. Molecular weight and isoelectric point were calculated by ExPASy (https://web.expasy.org/protparam/) [44].

### Phylogenetic analysis

The known CDK amino acid sequences in the *Homo sapiens* and *Mus musculus* were downloaded from the UniProt database (https://www.uniprot.org/) (Additional file 2). These, together with the identified and known amino acid sequences of CDKs in *Bos taurus*, *Bos grunniens*, *Hybrid-Bos indicus*, *Hybrid-bos taurus*, *Bos mutus*, *Bison bison bison*, *Bos indicus* and *Bubalus bubalis*, were aligned by ClustalW and a Neighbor-Joining tree was constructed in MEGA 7.0 [45]. The bootstrap was set as 1000 replicates. FigTree software (version 1.4.3) was used to adjust and beautify the evolutionary tree.

### Structural features analysis

To further evaluate the structural diversity of cattle CDK genes and proteins, a phylogenetic Neighbor-Joining tree was constructed. The conserved motifs were detected in MEME 5.0 [46] and visualized in TBtools [47]. The minimum and maximum number of amino acids in each motif was 6 and 50, respectively. The number of motifs in each CDK protein was limited to 10. Coding sequences and corresponding genomic sequences of bovine CDK genes were loaded into TBtools to portray the number and positions of CDSs and introns graphically.

### Chromosomal distribution and collinearity analysis

Positional information of predicted CDK genes in *Bos taurus*, *Bos grunniens*, *Hybrid-Bos indicus*, *Hybrid-bos taurus*, *Bos indicus* and *Bubalus bubalis* was extracted from the genomic sequence and annotation files and then was visualized in TBtools [47]. The identified CDKs in each species were mapped onto chromosomes. Comparisons between pairs of genomes were performed by all-against-all BLASTP searches (e-value = 10^**−** 5^) using the proteome sequences of *Bos taurus* as queries against those of the other five bovine species above. Collinearity analysis of ortholog genes between *Bos taurus* and the other five species was conducted using the MCScanX toolkit [48]. Results of collinearity analysis and orthologous CDKs were visualized by TBtools [47].

### Gene expression analysis by RNA-seq

RNA-Seq data of preadipocytes and differentiated adipocytes was downloaded from the National Center for Biotechnology Information (NCBI) Sequence Read Archive (accession number SRP067820) [49] and transformed into fastaq format by Fastq-dump. The sequencing quality was checked using FastQC [50]. Quality control of raw sequence data, including removal of the adapter sequences and low-quality sequences was performed using the Trim_galore [51]. Clean reads were then mapped to the *Bos taurus* genome (ARS-UCD1.2.101) using STAR [52]. RSEM [53] and FeatureCounts [54] were used to calculate the expression of the transcripts. Data was normalized by calculating the RPKM for each gene. The limma package [55] in R was used to analyze the differential expression under the threshold of fold change ≥1.2 and *p*-value < 0.05. The Sequence Read Archive Run (SRR) number and adjusted RPKM values of 163 tissue samples were downloaded from the Ruminant Genome Database (http://animal.nwsuaf.edu.cn/code/index.php/Ruminantia) [56] (Additional file 8). The heat map was constructed using the pheatmap package in R software [57].

### Isolation, culture and induction differentiation of bovine primary adipocytes

Primary adipocytes were isolated and cultured from perirenal adipose tissue of premature calf in the Zerui ecological breeding farm. Type I collagenase digestion method was used for the isolation and cultivation of calf preadipocytes. Induction of preadipocytes differentiation [58] and Oil Red O staining [59] were performed as described. Images were captured using a 10× objective lenses without filter model on an OLYMPUS IX50 microscope (Olympus Corporation, Tokyo, Japan) equipped with a charge-coupled device (CCD) camera (Diagnostic Institute, Inc., Sterling Height, MI, USA). CellSens V3.2 acquisition software was used and the images with a resolution at 96 dpi were acquired.

### RNA extraction and quantitative RT-PCR (qPCR)

The primers of the CDK genes were designed using Primer Premier 5.0 software (Additional file 9). Total RNA was extracted by the phenol-chloroform method using TRIzol (9109, Takara), and bovine adipocytes (*n* = 3) during the differentiation of 0, 2, 4, 6 and 10 days were used. Absorbance was measured at 260 nm and 280 nm and samples with an OD260/OD280 ratio between 1.8 and 2.0 were used in subsequent experiments. Then, 1000 ng total RNA was reverse transcribed using random primers with Moloney murine leukemia virus reverse transcriptase (Takara Bio, Kyoto, Japan). Realtime PCR was carried out in a CFX96 Touch Real-Time PCR Detection System (Bio-Rad, Hercules, CA, USA) with SYBR Green Master Mix (Takara Bio, Kyoto, Japan). The qPCR reaction procedure was 40 cycles of pre-denaturation for 3 min (95 °C), denaturation for 10 s (95 °C), annealing for 20 s (59–61 °C), extension for 30 s (72 °C). Glyceraldehyde-3-phosphate dehydrogenase (GAPDH) was selected as the reference gene.

### Statistical analysis

All qRT-PCR results were determined using the 2^−∆∆Ct^ method [60, 61]. Three independent technical repetitions were performed for each test. Statistical significance determined using Graphpad Prism 7.0 software.

## Supplementary Information

**Additional file 1.** Genome-wide identified CDK family members in Bovidae.

**Additional file 2.** Protein sequences of CDK family members in ten species.

**Additional file 3.** Conserved domain prediction of bovine CDK protein sequences.

**Additional file 4.** Amino acid sequences logos of 10 identified motifs in bovine CDK proteins.

**Additional file 5.** Oil Red O staining of preadipocytes and differentiated adipocytes.

**Additional file 6 **Conserved domain and motifs of *hybrid-Bos taurus* CDK proteins.

**Additional file 7.** The interaction network for CDK genes constructed by Cytoscape.

**Additional file 8.** Expression values (RPKM) of 163 bovine samples in 60 tissue types.

**Additional file 9.** Primers sequences of 25 bovine CDK genes.

## Data Availability

The datasets analysed during the current study are available in the NCBI Short Read Archive (SRA) repository, [accession number SRP067820; web links for SRP067820: https://trace.ncbi.nlm.nih.gov/Traces/study/?acc=SRP067820&o=acc_s%3Aa; direct web links for SRR3056892 (https://sra-downloadb.be-md.ncbi.nlm.nih.gov/sos2/sra-pub-run-7/SRR3056892/SRR3056892.1), SRR3064490 (https://sra-downloadb.be-md.ncbi.nlm.nih.gov/sos2/sra-pub-run-7/SRR3064490/SRR3064490.1), SRR3064491 (https://sra-downloadb.be-md.ncbi.nlm.nih.gov/sos2/sra-pub-run-7/SRR3064491/SRR3064491.1), and SRR3064492 (https://sra-downloadb.be-md.ncbi.nlm.nih.gov/sos2/sra-pub-run-7/SRR3064492/SRR3064492.1)]. The web links for genome and annotation of *Bos taurus* are available in http://ftp.ensembl.org/pub/release-103/fasta/bos_taurus/dna/Bos_taurus.ARS-UCD1.2.dna.toplevel.fa.gz and http://ftp.ensembl.org/pub/release-103/gff3/bos_taurus/Bos_taurus.ARS-UCD1.2.103.gff3.gz; the web links for genome and annotation of *Bos grunniens* are available in http://ftp.ensembl.org/pub/release-103/fasta/bos_grunniens/dna/Bos_grunniens.LU_Bosgru_v3.0.dna.toplevel.fa.gz and http://ftp.ensembl.org/pub/release-103/gff3/bos_grunniens/Bos_grunniens.LU_Bosgru_v3.0.103.gff3.gz; the web links for genome and annotation of *Hybrid-bos taurus* are available in http://ftp.ensembl.org/pub/release-103/fasta/bos_taurus_hybrid/dna/Bos_taurus_hybrid.UOA_Angus_1.dna.toplevel.fa.gz and http://ftp.ensembl.org/pub/release-103/gff3/bos_taurus_hybrid/Bos_taurus_hybrid.UOA_Angus_1.103.gff3.gz; the web links for genome and annotation of *Hybrid-Bos indicus* are available in http://ftp.ensembl.org/pub/release-103/fasta/bos_indicus_hybrid/dna/Bos_indicus_hybrid.UOA_Brahman_1.dna.toplevel.fa.gz and http://ftp.ensembl.org/pub/release-103/gff3/bos_indicus_hybrid/Bos_indicus_hybrid.UOA_Brahman_1.103.gff3.gz; the web links for genome and annotation of *Bos mutus* are available in http://ftp.ensembl.org/pub/release-103/fasta/bos_mutus/dna/Bos_mutus.BosGru_v2.0.dna.toplevel.fa.gz and http://ftp.ensembl.org/pub/release-103/gff3/bos_mutus/Bos_mutus.BosGru_v2.0.103.gff3.gz; the web links for genome and annotation of *Bison bison bison* are available in http://ftp.ensembl.org/pub/release-103/fasta/bison_bison_bison/dna/Bison_bison_bison.Bison_UMD1.0.dna.toplevel.fa.gz and http://ftp.ensembl.org/pub/release-103/gff3/bison_bison_bison/Bison_bison_bison.Bison_UMD1.0.103.gff3.gz; the web links for genome and annotation of *Homo sapiens* are available in http://ftp.ensembl.org/pub/release-103/fasta/homo_sapiens/dna/Homo_sapiens.GRCh38.dna.toplevel.fa.gz and http://ftp.ensembl.org/pub/release-103/gff3/homo_sapiens/Homo_sapiens.GRCh38.103.gff3.gz; the web links for genome and annotation of *Mus musculus* are available in http://ftp.ensembl.org/pub/release-103/fasta/mus_musculus/dna/Mus_musculus.GRCm39.dna.toplevel.fa.gz and http://ftp.ensembl.org/pub/release-103/gff3/mus_musculus/Mus_musculus.GRCm39.103.gff3.gz; the web links for genome and annotation of *Bos indicus* are available in https://ftp.ncbi.nlm.nih.gov/genomes/all/GCF/000/247/795/GCF_000247795.1_Bos_indicus_1.0/GCF_000247795.1_Bos_indicus_1.0_genomic.fna.gz and https://ftp.ncbi.nlm.nih.gov/genomes/all/GCF/000/247/795/GCF_000247795.1_Bos_indicus_1.0/GCF_000247795.1_Bos_indicus_1.0_genomic.gff.gz; and the web links for genome and annotation of *Bubalus bubalis* are available in https://ftp.ncbi.nlm.nih.gov/genomes/all/GCF/003/121/395/GCF_003121395.1_ASM312139v1/GCF_003121395.1_ASM312139v1_genomic.fna.gz and https://ftp.ncbi.nlm.nih.gov/genomes/all/GCF/003/121/395/GCF_003121395.1_ASM312139v1/GCF_003121395.1_ASM312139v1_genomic.gff.gz. The accession numbers listed in Table 1 and Additional file 1 can be referred in the Ensembl database (http://asia.ensembl.org/index.html) and/or NCBI Gene repository (https://www.ncbi.nlm.nih.gov/gene/). All the other data generated or analyzed during this study are included in this published article and its supplementary information files.

## Abbreviations

CDK: Cyclin-dependent kinases
MCE: Mitotic clonal expansion
CDS: Coding sequences
HMM: Hidden Markov Model
Chr: Chromosome
IMF: Intramuscular fat
pI: Isoelectric points
Mw: Molecular weight
