# Supplementary figures and images for: Genome-wide identification of cyclin-dependent kinase (CDK) genes affecting adipocyte differentiation in cattle
Source: BMC Genomics. 2021 Jul 12;22:532. doi: 10.1186/s12864-021-07653-8 (PMC8276410; doi:10.1186/s12864-021-07653-8)

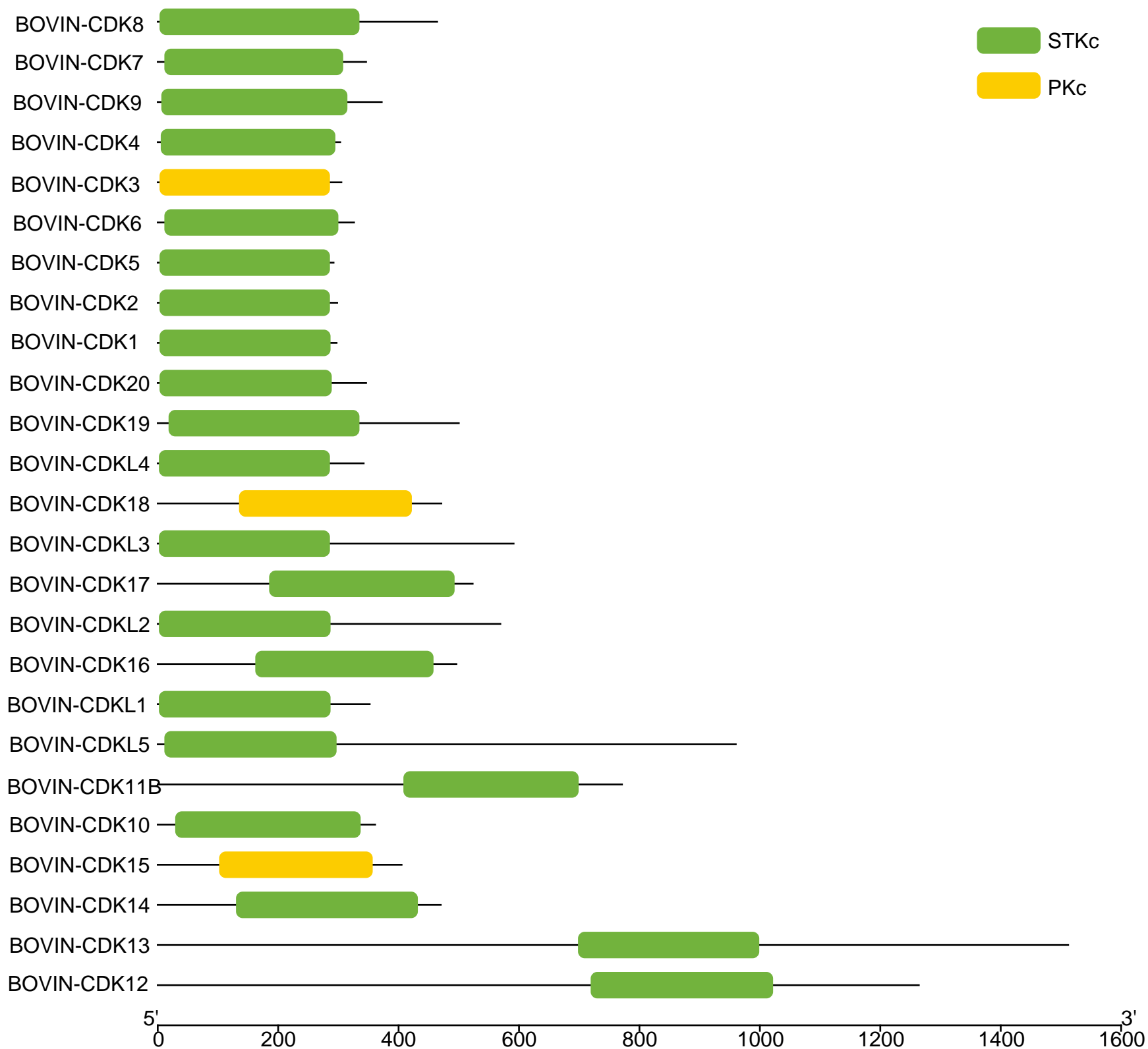

Supplement: Supplementary file 3 — Additional file 3. Conserved domain prediction of bovine CDK protein sequences. [file 12864_2021_7653_MOESM3_ESM.pdf]

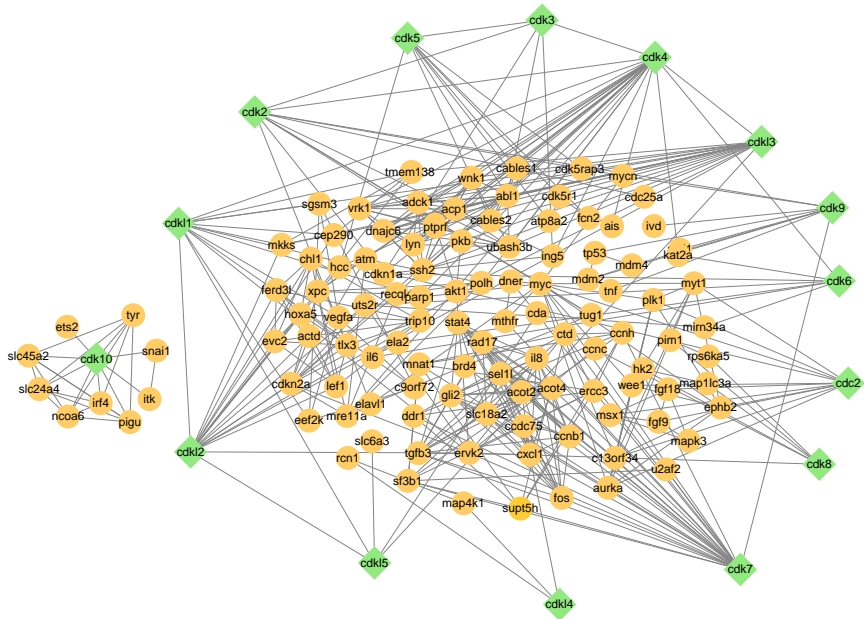

Supplement: Supplementary file 7 — Additional file 7. The interaction network for CDK genes constructed by Cytoscape. [file 12864_2021_7653_MOESM7_ESM.pdf]
