# Supplementary material for: Genome-wide identification of cyclin-dependent kinase (CDK) genes affecting adipocyte differentiation in cattle
Source: BMC Genomics. 2021 Jul 12;22:532. doi: 10.1186/s12864-021-07653-8 (PMC8276410; doi:10.1186/s12864-021-07653-8)
Supplement: Supplementary file 4 — Additional file 4. Amino acid sequences logos of 10 identified motifs in bovine CDK proteins. [file 12864_2021_7653_MOESM4_ESM.pdf]

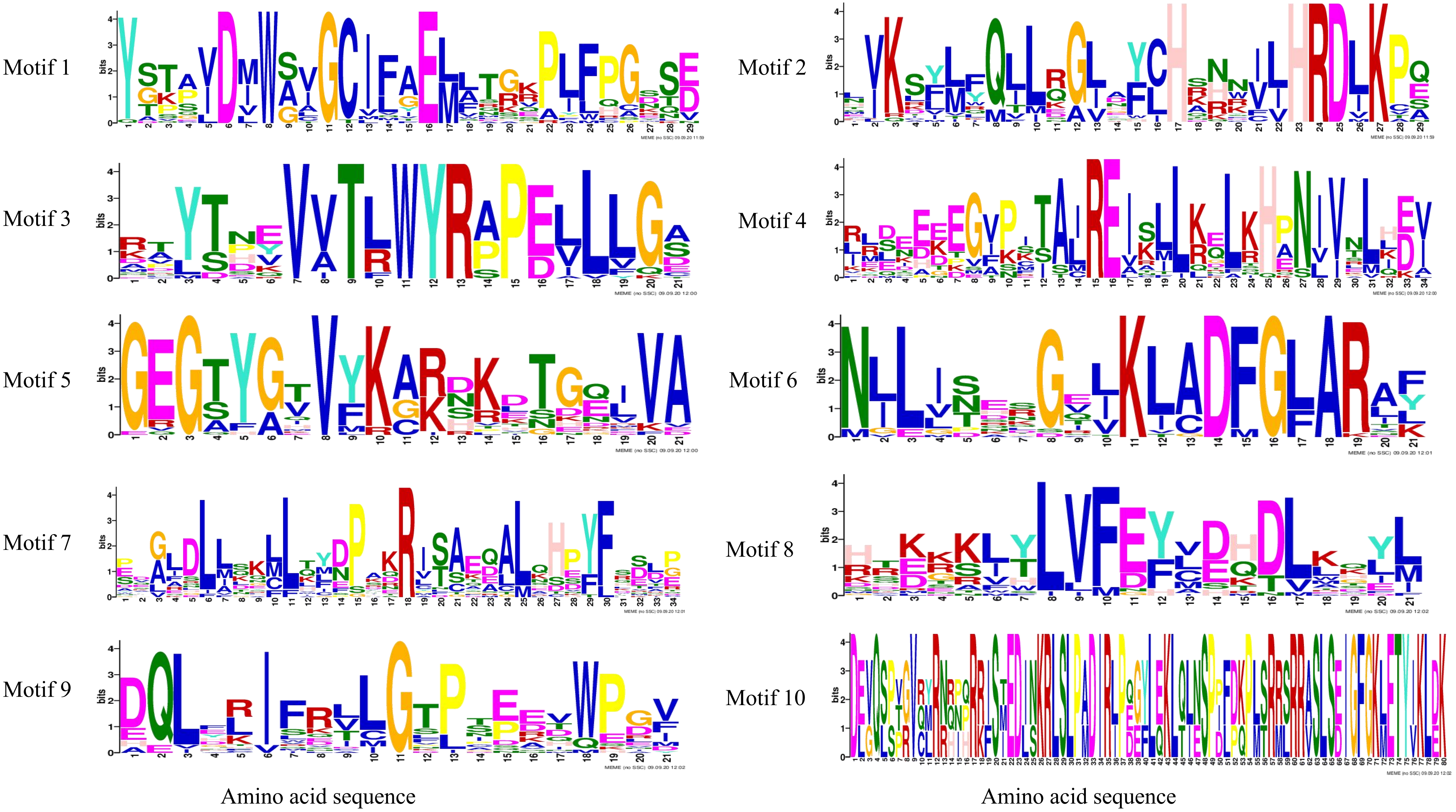
Motif 1
Motif 2
Motif 3
Motif 4
Motif 5
Motif 6
Motif 7
Motif 8
Motif 9
Motif 10
bits
Amino acid sequence
Amino acid sequence
