# Supplementary material for: Genome-wide identification of cyclin-dependent kinase (CDK) genes affecting adipocyte differentiation in cattle
Source: BMC Genomics. 2021 Jul 12;22:532. doi: 10.1186/s12864-021-07653-8 (PMC8276410; doi:10.1186/s12864-021-07653-8)
Supplement: Supplementary file 5 — Additional file 5. Oil Red O staining of preadipocytes and differentiated adipocytes. [file 12864_2021_7653_MOESM5_ESM.pdf]

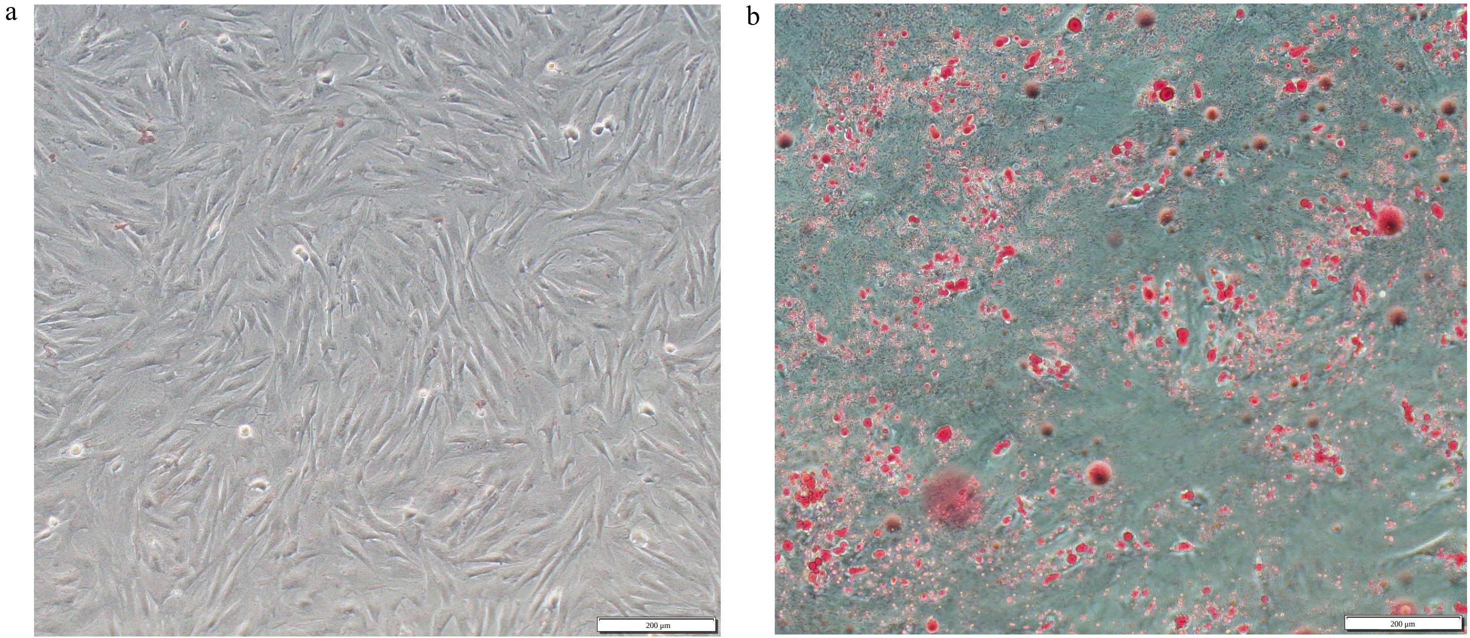

Images of Oil Red O staining in cattle adipocytes induced at day 0 (a) and day 10 (b) of adipogenic difffferentiation. Scale bar, 200 µm. Resolution, 9 6   d p i .
