# Supplementary material for: Genome-wide identification of cyclin-dependent kinase (CDK) genes affecting adipocyte differentiation in cattle
Source: BMC Genomics. 2021 Jul 12;22:532. doi: 10.1186/s12864-021-07653-8 (PMC8276410; doi:10.1186/s12864-021-07653-8)
Supplement: Supplementary file 6 — Additional file 6 Conserved domain and motifs of hybrid-Bos taurus CDK proteins. [file 12864_2021_7653_MOESM6_ESM.pdf]

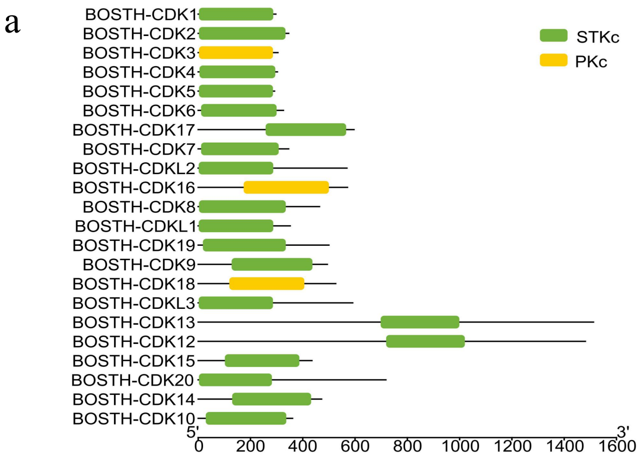

Conserved domain prediction of hybrid-*bos taurus* CDK proteins

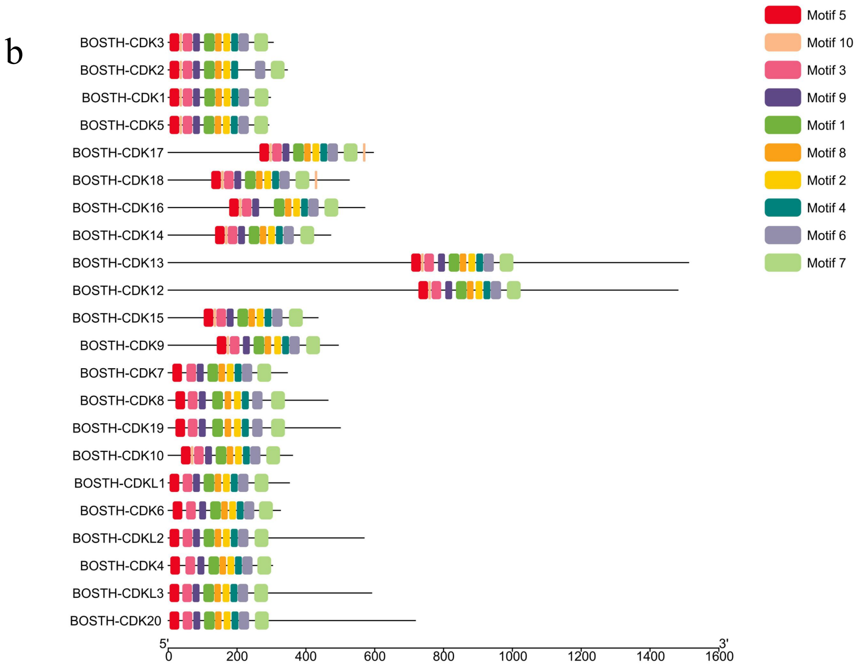

Conserved motifs prediction of hybrid-*bos taurus* CDK proteins
